# Physiochemical Characterization of Briquettes Made from Different Feedstocks

**DOI:** 10.1155/2012/165202

**Published:** 2012-06-27

**Authors:** C. Karunanithy, Y. Wang, K. Muthukumarappan, S. Pugalendhi

**Affiliations:** ^1^Department of Agricultural and Biosystems Engineering, South Dakota State University, Brookings, SD 57007, USA; ^2^Department of Bioenergy, Tamil Nadu Agricultural University, Coimbatore 641003, India

## Abstract

Densification of biomass can address handling, transportation, and storage problems and also lend itself to an automated loading and unloading of transport vehicles and storage systems. The purpose of this study is to compare the physicochemical properties of briquettes made from different feedstocks. Feedstocks such as corn stover, switchgrass, prairie cord grass, sawdust, pigeon pea grass, and cotton stalk were densified using a briquetting system. Physical characterization includes particle size distribution, geometrical mean diameter (GMD), densities (bulk and true), porosity, and glass transition temperature. The compositional analysis of control and briquettes was also performed. Statistical analyses confirmed the existence of significant differences in these physical properties and chemical composition of control and briquettes. Correlation analysis confirms the contribution of lignin to bulk density and durability. Among the feedstocks tested, cotton stalk had the highest bulk density of 964 kg/m^3^ which is an elevenfold increase compared to control cotton stalk. Corn stover and pigeon pea grass had the highest (96.6%) and lowest (61%) durability.

## 1. Introduction

In the last four decades, researchers have been focusing on alternate fuel resources to meet the ever-increasing energy demand and to avoid dependence on crude oil. Biomass appears to be an attractive feedstock because of its renewability, abundance, and positive environmental impacts resulting in no net release of carbon dioxide and very low sulfur content. Biomass is very difficult to handle, transport, store, and utilize in its original form due to factors that can include high moisture content, irregular shape and sizes, and low bulk density. Densification can produce densified products with uniform shape and sizes that can be more easily handled using existing handling and storage equipment and thereby reduce cost associated with transportation, handling, and storage. Tumuluru et al. [1] classified conventional biomass densification processes into baling, pelleting, extrusion, and briquetting, which are carried out using a bailer, pelletizer, screw press, piston press, or roller press. Baling, briquetting, and pelleting are the most common biomass densification methods; pelleting and briquetting are the most common densifications used for solid fuel applications.

In general, biomass/feedstock is a cellular material of high porosity since cells interior consists mainly of large vacuole-filled air in dry conditions [2]. In general, natural binders such as lignin, protein, and starches present in the feedstocks enhance the bonding between particles during densification process. Because of the application of high pressures, particles are brought close together, causing inter-particle attraction forces, and the natural binding components in the feedstocks are squeezed out of the cells, which make solid bridges between the particles [3]. Many feedstocks, densification machines, and process variables affect the quality of densified products. Several researchers have reported that feedstock composition such as lignin, hemicellulose, and extractives, types of feedstock, fraction of the same feedstock, feedstock particle size and moisture content, percentage of fines, type of densification machine, die diameter, preheating/steam injection, temperature, and pressure are the major variables that contribute to the quality of densified materials [4–12]. Feedstock composition is one of the major variables; therefore, understanding the compositional changes due to densification can be useful in understanding their compaction behavior [1].The Literature survey revealed that only Theerarattananoon et al. [13] reported the changes in chemical composition before and after pelleting different feedstocks, none on briquetting. The dimensions of pellet, friction/shear development during pelleting, and briquetting would be different. Hence, this study was undertaken with two objectives: (1) to study the changes in chemical composition of different feedstocks due to briquetting and (2) to validate the relation of different variables that contribute to bulk density and durability.

## 2. Materials and Methods

### 2.1. Feedstocks Preparation and Characterization 

Switchgrass and prairie cord grass obtained from different local farms were ground in hammer mill (Speedy King, Winona Attrition Mill Co, MN) using an 8 mm sieve for densification and sent to Tamil Nadu Agricultural University (TNAU), Coimbatore, India. Similarly, corn stover, pigeon pea grass, and cotton stalk were collected from experimental field at TNAU, Coimbatore, India. Sawdust was obtained from local sawmill located at Coimbatore, India. The compositional analyses of the feedstocks and briquettes such as total solids, cellulose, hemicellulose, lignin, ash, and extractives content were carried out in triplicate as outlined by Sluiter et al. [14–16] using muffle furnace and HPLC and reported in Table 1. 

### 2.2. Particle Size Analysis 

Prior to briquetting, the geometric mean diameter of ground feedstocks was determined using ASAE Standard S319.4 [17] with the help of a Ro-Tap sieve shaker (W. S. Tyler Inc., Mentor, OH, USA) with US sieve numbers 6, 7, 10, 16, 20, 30, 50, 70 100, 140, 200, and 325 (sieve opening sizes: 3.35, 2.80, 2.00, 1.190, 0.841, 0.595, 0.297, 0.210, and 0.149 mm, resp.). For each test, a 100 g sample was placed on a stack of sieves arranged from the largest to the smallest opening. A 10-minute sieve shaking time was used as mentioned in the ASAE Standard S319. The geometric mean diameter (dgw) of the sample and geometric standard deviation of particle diameter (Sgw) were calculated in replicates of three for each feedstock. 

#### 2.2.1. Briquetting 

**Figure 1:**
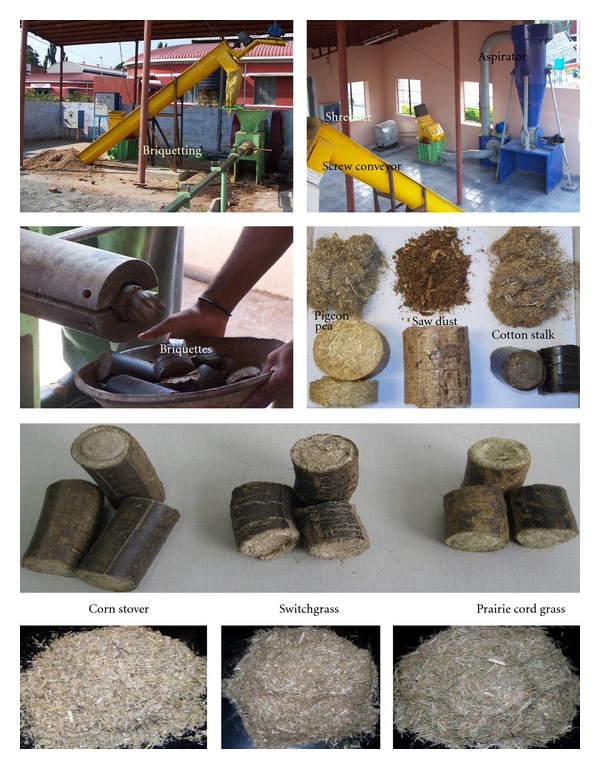
Briquetting system along with control and briquettes.

The briquetting system consists of 40 hp motor, feed hopper, and die section, and the capacity is 150–200 kg/h. The system had a provision to select 60 or 90 mm die section. For this study, 60 mm die was used.  Figure 1 shows the briquetting system along with feedstocks and briquettes. The briquetting machine is the simple horizontal briquetting press. Material handling screw conveyor with 2 hp electric motor coupled with reduction gear and variable pulley with V belt. The shaft moves an eccentric disc through connecting rod where circular motion is connected to linear motion. The eccentric disc is connected to an alloy steel piston which is having to-and-fro movement in stationary cast iron cylinder. The piston carries a hardened and ground alloy steel punch. The hardened ground alloy steel die is held in steel die holder. The raw material, passed into hopper of the machine, is transferred to a chamber where punch pushes the material into the die, forms the cylindrical briquette, and pushes it further into split die and cooling line. Briquettes were collected and sent through FedEx to South Dakota State University for further analysis.

#### 2.2.2. Density and Porosity

Bulk densities of ground feedstocks and briquettes were measured following the ASAE standard method S269.4 DEC01 [18]. The container used is a 2000 mL glass container. The bulk density was calculated from the mass of feedstocks and briquettes that occupied the container. 

The Micromeritics Multivolume Pycnometer and cell (125 cm^3^) provided with the equipment was used for the measurement of the true density of the samples. The measurement is based on the pressure difference between a known reference volume and the volume of the sample cell. Helium is used as the gas to fill the reference and sample cells at 19.5 ± 0.2 psi as specified in the instrument manual. True density of the material was measured using equation

(1)True density=m{Vcell−Vexp⁡/[(P1/P2)−1]},

where *m* is the weight of the sample, *V*_cell_ is empty volume of the sample cell, **V*_exp⁡_* is expansion volume, *P*1 is pressure before expansion, and *P*2 is the pressure after expansion.

Porosity is a measure of the void spaces in a material and is a fraction of the volume of voids over the total volume; it generally lies between 0-1. The porosity is calculated by the true density and bulk density measured as explained previously:

(2)Porosity=(1−Bulk densityTrue density).

### 2.3. Durability

The durability of the briquettes was determined using a pellet durability tester (model PDT-110, Seedburo Equipment Company, Chicago, IL) following method S269.4 [18]. About 200 g of briquettes were divided into two batches of 100 g each. Each batch was placed in the pellet durability tester for a period of 10 min and operated at 50 rpm. The sample was placed on a no. 4 sieve (4.75 mm) before and after tumbling and measured for the mass retained on the screen. The pellet durability was then calculated using the following equation:

(3)Durability=(MatMbt),

where **M*_at_* is the mass of the briquettes retained on the screen after tumbling (g), and *M*_bt_ is the mass of the briquettes retained on the screen before tumbling (g).

### 2.4. Glass Transition Temperature

The glass transition temperature (**T*_g_*) of the feedstocks was evaluated using a differential scanning calorimeter (DSC) (Q series, TM Model Q200, TA Instruments, New Castle, DE). A refrigerated cooling system (RCS40), provided with DSC module, has the ability to control the sample temperature from −40°C to 400°C. About 2.0–2.2 mg feedstock was in **T*_zero_* aluminum pan and subjected to a heating range of 10 to 150°C with a heating rate of 5°C/min. An empty *T*_zero_ aluminum pan was considered the reference cell. Universal analyzer software provided by TA instruments (New Castle, DE) was used to analyze **T*_g_* from the thermograms, using the half-height integration method [19]. 

### 2.5. Statistical Analysis

All physical and chemical properties measurements were made in triplicate, and the data were analyzed with Proc GLM procedure to determine the statistical significance using SAS 9.2 [20] using a type I error (*α*) of 0.05.

## 3. Results and Discussion

Briquetting machines can handle larger particles with wide range of moisture content without additional binders, not the pellet mills. Further, friction/shear between the particles and the briquetting machine is much less than that of pelleting/cubing [21]. The standard shape of a fuel pellet is cylindrical, with a diameter of 6 to 8 mm and a length of no more than 38 mm. If the pellets are with more than 25 mm in diameter, they are usually referred to as “briquettes.” The dimensions of the pellets found in the literatures are 4–7 mm diameter and 13–23 mm length [22, 23], whereas briquettes can have diameter between 25 and 100 mm with length between 25 and 280 mm [24]. The dimensions, friction/shear, steam injection/preheating, and binder would make much more differences in the resultant compacts, which should be considered to compare the briquettes data presented in this study.

### 3.1. Particle Size Analysis

**Figure 2:**
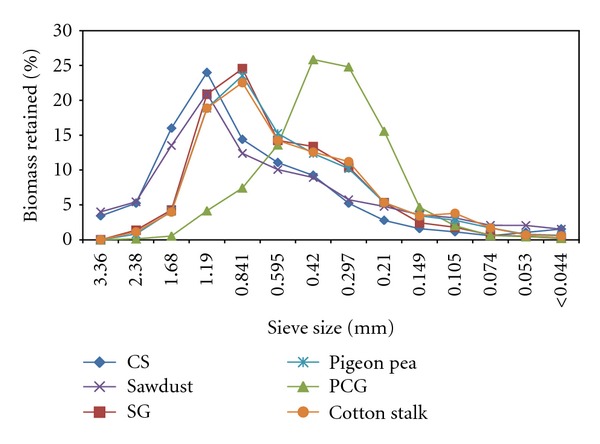
Particle size distribution of different feedstocks used in this study.

Apart from moisture content, particle size distribution and particle size are two important factors that affect the bulk physical properties of feedstocks. Bulk density of ground feedstocks depends on the particle size and particle size distribution. Particle size distribution also reflects on the available surface area. Particle sizes affect the true density of the feedstocks [25] and also influence durability [9]. Particle size analyses of the feedstocks are shown in Figure 2. In general, all the feedstocks had more than 50% of the particle size in the range of 0.297–1.68 mm as evident from the figure. A major fraction of the PCG was shifted towards lower particle size because of their needle-like shape (Figure 1 PCG control). Switchgrass, pigeon pea grass, and cotton stalk had a similar distribution as evident from Figure 2. Though different screen/sieves were used during grinding, similar trend of particle size distribution (normal distribution) was reported for switchgrass [26], olive tree pruning [12], barley, canola, oat, and wheat straws [27]. Colley et al. [26] reported that sieves with aperture sizes of 0.595 and 0.850 mm retained 29.5 and 38.6% switchgrass ground using 3.18 mm screen; in this study, 8 mm screen is used for grinding which explains the difference in particle retention recorded. Sawdust particle distribution was different from Rhén et al. [7] where they dried and milled the sawdust using 4 mm sieve; hence, they could get particles less than 0.5 mm about 44%. 

The percentage of fines has influence during densification. In general, fines would result in more durable product, and it comes with grinding cost, which is not desirable. In general, the finer the feedstock grinds, the higher the quality of compact [9]. Tabil and Sokhansanj [28] considered that particles with sizes below 0.400 mm are fine and highly compressible. Taking this criterion into account, PCG had a maximum fine of 48.3%, followed by cotton stalk (26.7%), and corn stover had the least (13.9%). Olive tree pruning had 18% fines when 4 mm screen was used [12], 14% fines from switchgrass when 3.18 mm screen was used [26], and more than 60% fines from barley, canola, oat, and wheat straws when 1.98 mm screen was used [27]. The differences in fines are mainly due to variation in screen sizes and inherent characteristics of the feedstocks. According to MacBain [29], large particles are fissure points that cause cracks and fractures in compacts. Further, large particles in compact mean inhomogeneous shrinking, which would develop cracks [5]. The cracks on the surface of the briquettes (Figure 1) might be due to larger particles. Several researchers have reported that mixture of different particle sizes would result in better quality due to interparticle bonding with no interparticle space [29, 30].

The order of geometrical mean diameter (GMD) was corn stover (0.833 mm), switchgrass (0.736 mm), sawdust (0.708 mm), pigeon pea grass (0.657 mm), cotton stalk (0.639 mm), and PCG (0.0432 mm), and their geometric standard deviation of particle diameter (Sgw) was 0.422, 0.300, 0.455, 0.341, 0.347, and 0.251 mm, respectively. For switchgrass, Colley et al. [26] recorded a high GMD of 0.867 mm with the geometric standard deviation of 0.357 mm when 3.18 mm screen used. Mani et al. [8] reported a lower GMD of 0.193–0.412 and 0.253–0.456 mm with geometric standard deviation of 0.261–0.447 and 0.255–0.438 mm, respectively, for corn stover and switchgrass ground using 0.8–3.2 mm screen. Similarly, Kaliyan and Morey [21] reported a lower GMD of 0.56–0.66 mm for corn stover and switchgrass when 3 mm screen was used for grinding. Adapa et al. [27] also reported lower GMD in the range of 0.347–0.398 mm for barely, canola, oat, and wheat straws. These differences are mainly due to variation in screen sizes used during grinding (0.8–3.2 versus 8 mm). In a recent study, when Adapa et al. [31] used screen size 6.4 mm, GMD of barely, canola, oat, and wheat straws was 0.883, 0.885, 0.935, and 0.997 mm, respectively. Though they used lower screen size (6.4 mm) than this study (8 mm), GMD was higher than the feedstocks used in this study and that might be due to inherent characteristics of the feedstocks. 

### 3.2. Moisture Content

**Figure 3:**
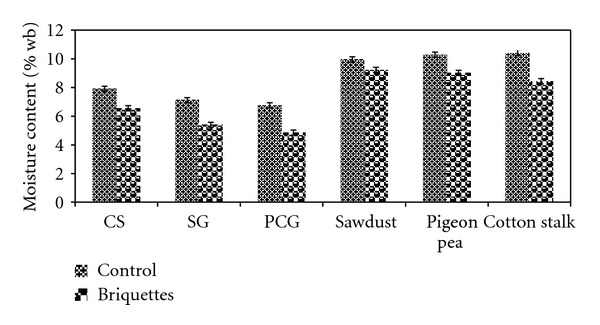
Effect of briquetting on moisture content.

Moisture content has strong influence on density, durability, and storage. Several researchers have recommended a range of moisture content for pelleting or briquetting of different feedstocks. Moisture content (wb) for pelleting pruning of olive residues would be less than 10% wb [12]: about 10% for switchgrass [10], about 8-9% for alfalfa [32], 6–12% for wood [33], and 5–10% for corn stover [34]. The moisture content of the feedstocks ranged between 6.8 and 10.4% wb, whereas it was 4.9–9.2% wb for briquettes as depicted in Figure 3; the values are well within the range of moisture content reported in the above literatures. The moisture content decrease is due to rise in feedstocks temperature during briquetting. Though PCG had the lowest moisture content, the highest decrease of 28% was observed. Similar observation was reported when Kaliyan and Morey [21] briquetted corn stover and switchgrass with feedstock moisture content in the range of 15–20% wb, the resulted briquettes had an average moisture content in the range of 11–14.5%, which was equivalent to 25–29% decrease in moisture. A minimum change in moisture content due to briquetting for sawdust, among the feedstocks is studied. In general, the compacted/densified biomass would have the moisture content between 7 and 14% [35], and the briquettes produced for this study had moisture content within the range that indicates better density, durability, and storage. 

### 3.3. Chemical Composition

The composition of feedstock is one of the major variables that contribute to the quality of briquettes/compacts/pellets. The feedstock has low-molecular-weight substances such as organic matter, inorganic matter, and macromolecular substances include cellulose, hemicellulose, and lignin [36]. Understanding the major compositional changes that take place during briquetting can be useful in understanding their compaction behavior [1]. Compositional analysis of the feedstock and briquettes is presented in Table 1. Because of moisture and volatile matters loss due to temperature rise during briquetting, the chemical compositions of briquettes change slightly. Significant change in glucose content was observed irrespective of the feedstocks. Among the feedstocks, PCG had a maximum of 17.5% increase in glucose, whereas cotton stalk had a decrease of 8.7%. For most of the briquettes, xylose content has decreased when compared to their respective feedstocks. Similarly lignin content of the briquettes was less than that of the feedstocks except sawdust. Recently, Theerarattananoon et al. [13] reported similar observation for glucose, xylose, and lignin of pellets produced from wheat straw, corn stover, big bluestem, and sorghum stalk. Briquettes made from PCG and sawdust have shown an increase in lignin. However, increase in lignin was not significant for PCG and it was in agreement with the findings of Theerarattananoon et al. [13]. The change in ash content is inconsistent across the feedstocks, and similar results were reported for different feedstocks [13]. When compared to other feedstocks, cotton stalk had high volatile matters that reflected on high smoke production as well as brown liquid oozed out during briquetting, thereby more changes in the chemical composition including ash content. According to Kaliyan and Morey [21], ash/mineral content of the feedstocks would show their relative abrasiveness to equipment when there is high friction/shear during densification; the higher the ash content, the higher the abrasion. Ash content of corn stover, pigeon pea grass, and cotton stalk briquettes increased significantly, whereas switchgrass and sawdust briquettes had a significant decrease. Kaliyan and Morey [21] have also reported similar ash/mineral contents of corn stover (11.2%) and switchgrass (5.0%). Ash content of the cotton stalk briquettes increased by 1.8 times. 

Extractive is the material present in the feedstock which is soluble in either water or ethanol during extraction and that is not an integral part of the cellular structure [16, 37]. Inorganic material, nonstructural sugars, and nitrogenous material are water soluble, whereas ethanol soluble includes chlorophyll, waxes, or other minor components. Nonstructural component refers to nonchemically bound components of the feedstock that include but are not limited to sucrose, nitrate/nitrites, protein, ash, chlorophyll, and waxes [16]. A mix of long-chain fatty acids, fatty alcohols, sterols, and alkanes are the main constituents of wax [38, 39]. The types of extractives found in the feedstocks are entirely dependent upon the feedstock itself [37]. In general, grasses contain higher amount of extractives than wood, and it can be observed in the Table 1. The change in extractives was not the same for all the feedstocks, and this observation was in agreement with Theerarattananoon et al. [13]. Corn stover and pigeon pea grass briquettes had significantly higher extractives than that of their respective feedstocks. Extractives of corn stover briquettes increased about 14%, which is similar to increase in extractives of corn stover pellet [13]. A maximum increase and decrease in extractives of 130 and 44%, respectively, were recorded for pigeon pea grass and sawdust briquettes. Higher percentages of extractives (waxes, resins, and starches) affect gluability, contribute to the reduction of shrinkage, and would increase the bonding and the overall pellet strength [4, 5]. 

### 3.4. Glass Transition Temperature (*T*_*g*_)

Lignin could be the deciding factor, and it has strong influence on binding characteristics, thereby the briquette and pellet quality [11, 27]. Lignin content varies depending upon the type of feedstocks [11] and between the fractions of the same feedstock [40]. As noted in Table 1, lignin content of sawdust differed from other feedstocks. According to Back and Salmen [41], lignin and hemicellulose undergo plastic deformation at temperature in their glass transition/softening temperatures. Softening temperature is of high importance, because many properties including elastic modulus would change remarkably when the material passes from a glassy into a rubbery state. The higher the temperature above the **T*_g_*, the greater and easier is the flow of these molecules [2]. Corn stover, switchgrass, and PCG had a glass transition temperature of 79.2, 82.5, and 80°C, respectively, and they are very close to each other and it was in agreement with the average glass transition temperature (75°C) of corn stover and switchgrass reported by Kaliyan and Morey [21]. Pigeon pea grass, saw dust, and cotton stalk had a glass transition temperature of 75, 72, and 82°C, respectively. Van Dam et al. [42] reported that lignin has low melting point (~140°C) and thermosetting properties that would help for active binding. The temperatures of the briquettes at the exit were in the range of 130–140°C and confirm that lignin would have crossed its glass transition and melting point. 

### 3.5. Bulk and True Densities

**Figure 4:**
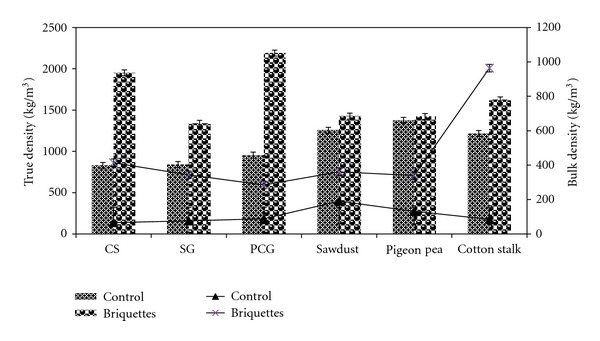
Effect of briquetting process on bulk and true densities.

Bulk density plays vital role in transportation and storage efficiency. In addition, bulk density influences the engineering design of transport equipment, storages, and conversion process [43]. Bulk and true densities of the feedstocks and briquettes are depicted in Figure 4. As noted from the figure, bulk density of the feedstocks ranged between 66 to 191 kg/m^3^, whereas the briquettes bulk density varied between 285–964 kg/m^3^. Among the feedstocks, corn stover had the lowest and sawdust had the highest bulk density. Bulk density of the feedstocks used in this study was well within the range reported for barely and oat straws by Adapa et al. [31]; though they used lower screen sizes (0.8–6.4 mm) during grinding. Mani et al. [8] have reported higher bulk density for corn stover (131–158 kg/m^3^) and switchgrass (115–182 kg/m^3^). Similarly, Kaliyan and Morey [21] also reported a higher bulk density of 103–160 and 181–220 kg/m^3^, respectively, corn stover and switchgrass. Possible reason for their higher bulk density is the use of lower screen sizes (0.8–3.2 and 2.4–4.6 mm) for grinding. Several researchers have reported that densification would result in bulk densities in the range of 450 to 700 kg/m^3^ depending upon feedstock and densification conditions [3, 21, 26, 44]. Among the briquettes, PCG and cotton stalk had the lowest and highest bulk density. Irrespective of the feedstocks, briquettes bulk density increased, which is one of the purposes of briquetting. The lowest increase of 1.9 times was observed for sawdust, and the highest increase of 11.3 times was noted for cotton stalk. Depending upon the briquetting machine used, the bulk density of feedstock would increase approximately 10–20 times of its original bulk density [35]. Except bulk density of cotton stalk, briquettes made from other feedstocks did not fall within the expected range, that is, 10–20 times increase. Possible reason might be the type of briquetting machine used, the feedstock properties, and the process conditions employed in this study. However, increase in bulk density of corn stover and switchgrass briquettes were higher than that of Kaliyan et al. [3] reported for corn stover (2.9–3.4 times) and switchgrass (1.6–2.3 times) depending upon the feedstock particle size and temperature, roller speed, and feeder screw speed. The increase in bulk density correlates well with porosity of the feedstocks as discussed in the next subheading. Though switchgrass pellets [26] had higher bulk density (536–708 kg/m3) than that of this study, but increase in bulk density was only threefold which was lower than that of this study (4.5 times). 

True density of the feedstocks varied between 830 and 1376 kg/m^3^, whereas it varied between 1340 and 2190 kg/m^3^ for briquettes as shown in Figure 4. An increase in true density was in the range of 1.03–2.35 times, whereas Adapa et al. [31] reported decrease in true density of pellets made from different feedstocks. A maximum and minimum increase in true density was observed for corn stover and pigeon pea grass, which is attributed to their structures. Because of lower screen sizes (0.8–3.2 mm) used for grinding, Mani et al. [8] have reported higher true density for corn stover (1170–1399 kg/m^3^) and switchgrass (946–1173 kg/m^3^). The true density of the feedstocks used in this study was in agreement with barely, canola, oat, and wheat straws [8, 31]. Though Kaliyan and Morey [21] used lower particle sizes of corn stover and switchgrass for briquetting, they reported true density of briquettes in the range of 825–1162 and 417–1065 kg/m^3^, respectively, depending on the pressure, preheating, feedstock particle size, and moisture content. 

As mentioned earlier, density and durability depends on many feedstocks, machines, and process variables. In order to have a comprehensive understanding of these variables, Table 2 is presented here. In general, bulk density of the briquette/pellet increased 2–13 times depending upon the feedstock, densification equipment, and process conditions. Corn stover briquette had higher true density than the studies listed in the table. True density of switchgrass briquette was lower than that of switchgrass pellet reported by Colley et al. [26] that might be the use of steam for raising the switchgrass grind temperature. Though Kaliyan and Morey [21] used preheating temperature of 25–150°C, their true density of switchgrass pellet was lower than that of the true density obtained in this study. Despite of the fact that Lehtikangas [5] used sawdust with less than 3 mm particle sizes, the true density was lower than that of sawdust true density obtained in the present study. In general, true density of briquettes produced in this study was higher than that of the values listed in Table 2. 

### 3.6. Porosity

**Figure 5:**
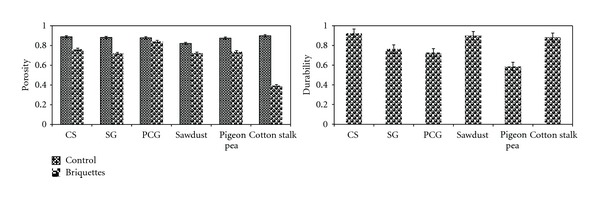
Effect of briquetting on porosity and durability.

Porosity has influence in transportation and storage. Porosity of the feedstocks and briquettes are presented in Figure 5. Sawdust and cotton stalk had the lowest (0.85) and highest (0.93) porosity among the feedstocks studied. Briquettes had lower porosity than that of their respective feedstocks. Among the feedstocks studied, a maximum of 56% decrease in porosity was recorded and that corresponds well with the bulk density. Cotton stalk briquettes had the lowest bulk density of 0.41 indicating less void space and more briquettes, which reflects on high bulk density (964 kg/m^3^). Low porosity of the feedstock which indicates that the void space is less and the feedstock within the given volume is more would result in low compressability, whereas high porosity would result in high compressability which was observed for sawdust and cotton stalk. Except cotton stalk briquette, briquettes made from other feedstocks had higher porosity than that of switchgrass pellets (0.516–0.626) [26]. This might be due to the differences in size or dimensions of briquettes and pellets.

### 3.7. Durability

Durability is a measure of the briquettes ability to withstand destructive forces such as compression, impact, and shear during handling and transportation. The production of fines or dust during handling, transport, and storage would create health hazard and inconvenient environment for the workers [50]. There is no limit for the production of fines in place. However, Dobie [51] suggested that fines up to 5% (by weight) would be an acceptable level, and greater than 5% would reduce storage capacity and create problems in flow characteristics. Depending upon the values, researchers have classified the durability into high (>0.8), medium (0.7-0.8), and low (<0.7) [6, 28]. Figure 5 shows durability of the briquettes that varied between 0.61 (PCG) and 0.97 (CS). High durability might be possible with larger particle size due to mechanical interlocking of relatively long fibers [52]. A noteworthy point is that these briquettes were produced in India and transported to Brookings, SD, USA through Fedex, wherein multiple handling has been involved, in spite of that, these briquettes had high durability. The differences in durability between briquettes might be due to chemical composition including lignin, extractive, cellulose, and hemicellulose, structure, fraction of leaf to stem or stalk, glass transition temperature, and compressibility. According to the above durability classification, corn stover, sawdust, and cotton stalk briquettes come under high, switchgrass and PCG briquettes fall under medium, and pigeon pea grass briquettes under low durability category. Kaliyan and Morey [21] reported a comparable durability of 0.50–0.97 for corn stover briquettes when corn stover grind size of 3 and 4.6 mm with a moisture content of 10–20% (wb) was preheated between 25 and 150°C and applied pressure in the range of 100–150 MPa. However, preheating temperature of 25°C resulted in low durability. Irrespective of switchgrass grind size, moisture content, pretreating temperature, and applied pressure, durability was in the range of 0–0.68 [21] that was lower than the durability of switchgrass briquettes produced in this study. Considering the moisture content of switchgrass, durability of switchgrass briquettes was in agreement with durability of switchgrass pellets [26]. The desirable durability of briquettes depends on the targeted use, that is, high durability for fuel application; low and medium durability would be sufficient for biochemical platform, because more surface area is desirable for enzymatic hydrolysis in biochemical platform. In addition, densification process would disturb the feedstock structure which would facilitate enzymatic hydrolysis. 

Overall, the durability of corn stover briquette is comparable to corn stover briquettes and pellets listed in Table 2. Durability of the switchgrass briquette was higher than that of switchgrass briquettes produced using preheating and pressure [21] and ultrasonic vibration-assisted switchgrass pelleting [49]. In general, durability of briquettes used in this study had been either high or comparable to briquettes and pellets made from different feedstocks as listed in the table. 

### 3.8. Correlation Analysis

In general, bulk density depends on chemical composition, particle size distribution, particle shape and size, orientation of particles, true density of individual particles, moisture content, and applied axial pressure [3, 7, 8, 21, 53–55]. Durability depends on the types of feedstock, fraction of different components (leaf, stem), lignin, extractives, particle size/GMD, fines, and moisture content apart from densification machine and process variables [3–12, 21]. Six different feedstocks were used for briquettes production in this study, and it would be appropriate to validate the relationship of different variables with bulk density and durability; accordingly correlation analysis was performed, and the coefficients are presented in Table 3. As noted in table, moisture and lignin contents had strong positive influence, whereas extractives had negative influence on bulk density of the briquettes. In general, lignin is heavier than extractives which would explain their contribution towards bulk density. Extractives and fines showed negative influence; lignin and GMD showed positive influence on durability of the briquettes. When lignin and extractives content exceed a threshold level of 34% in wood samples, pellet durability decreased [56]. Considering this threshold level, the correlation analysis showed a poor relation (*r* = 0.08). Since moisture contents of the briquettes were in a small range (4.9–9.2% wb), the relation with durability might not be prominent. This correlation analysis reveals that there is a need to determine the threshold level of each variable with respect to density and durability.

## 4. Conclusions

Briquettes were produced from variety of feedstocks to compare their physical and chemical properties. Statistical analyses revealed the existence of significant changes in chemical compositions, differences in density, porosity, and durability. Correlation analyses confirmed the contribution of lignin, extractives, fines, and particle size towards durability. This study confirms that medium size feedstock with low moisture content and lignin content in the range of 21–25% would result in 2–11-fold increase in density with medium and high durability. Cotton stalk briquettes had a bulk density of 964 kg/m^3^ with a durability of 0.923. 

## Figures and Tables

**Table 1:** Changes in chemical composition (%) due to briquetting.

	Glucose	Xylose	Lignin	Ash	Extractives
Control					

CS	36.0^f^	15.3^c^	22.4^d^	10.9^c^	11.3^e^
SG	31.2^g^	19.5^a^	24.7^c^	5.6^d^	18.5^b^
PCG	31.5^g^	15.5^bc^	21.4^d^	5.6^d^	20.3^a^
Sawdust	39.1^e^	10.5^f^	33.6^a^	5.3^d^	7.5^f^
Pigeon pea	50.3^a^	10.8^f^	24.2^c^	3.2^f^	6.1^g^
Cotton stalk	42.5^d^	16.5^b^	24.4^c^	5.2^d^	6.3^g^

Briquettes					

CS	38.4^e^	10.1^gf^	21.9^d^	11.9^b^	12.9^d^
SG	36.0^f^	19.0^a^	24.8^c^	3.7^ef^	16.5^b^
PCG	37.0^ef^	12.0^e^	22.5^d^	5.3^d^	17.0^b^
Sawdust	44.8^c^	13.3^d^	39.1^b^	3.9^e^	4.2^h^
Pigeon pea	47.3^b^	9.2^g^	21.0^d^	4.2^e^	13.9^c^
Cotton stalk	38.8^e^	14.8^c^	22.2^d^	14.6^a^	6.2^g^

Different letters within the same column indicates that means are statistically different (*P* < 0.05).

**Table 2:** Effect of briquetting and pelleting conditions of different feedstocks on bulk density, true density, and durability.

Densification	Process conditions	Feedstock conditions	Feedstock bulk density kg/m^3^	Product bulk density, kg/m^3^	True density, kg/m^3^	Durability	Reference
Briquetting	15.7–31.4 MPa; 80–105^°^C	Soda weed, 7–13% wb, < 10 mm	172	600–950	NR	NR	[45]
Hydraulic press pelletizer	55.2–552.3 bar, 50–125^°^C	Switchgrass, 6.5% wb, 10–70 mm	NR	250–720	NR	0.98–0.99	[10]
Sprout Matador 12 press pelletizer		Sawdust, <3 mm	NR	606–641	1228–1234	0.80–0.90	[5]
Modified SPC PP300 Compact pelletizer	0–2.6% steam addition, die temperature 83^°^C	Reed canary grass, 4 mm, 14–17% wb, precompacted to 269–356 kg/m^3^	140–160	600–700	NR	0.92–0.98	[46]
Single pelletizer	95^°^C, 30–134 MPa	Barley straw, <1.9 mm, 10% wb	261	NR	907–988	NR	[27]
Single pelletizer	95^°^C, 30–134 MPa	Canola straw, <1.9 mm, 10% wb	273	NR	823–1003	NR	[27]
Single pelletizer	95^°^C, 30–134 MPa	Oat straw, <1.9 mm, 10% wb	268	NR	849–1011	NR	[27]
Single pelletizer	95^°^C, 30–134 MPa	Wheat straw, <1.9 mm, 10% wb	269	NR	813–931	NR	[27]
Lab-scale CPM CL-5 pellet mill	Steam at 118^°^C,	Sun-cured and dehydrated alfalfa, 1.98–3.2 mm, 7–9.3%	NR	NR	1181–1341	0.43–0.92	[22]
Glomera extrusion press	5.7–8.3 MPa, 28.9–49.4^°^C	Wheat straw, 6–25 mm, 8.1–17.8%wb	NR	NR	1056	0.99	[47]
Glomera extrusion press	3.5–9.0 MPa, 27.8–59.4^°^C	Flax straw, 6–25 mm, 9.4–19% wb	NR	NR	1069–1260	0.99	[47]
Glomera extrusion press	3.1–9.6 MPa, 36.7–60^°^C	Sunflower stalk, 6–25 mm, 9–19% wb	NR	NR	940–1620	0.99	[47]
Lab-scale CPM CL5 pellet mill	Steam injection	Switchgrass, <3.18 mm, 6.3–17% wb	169.5	536–708	1410–1430	0.89–0.96	[26]
Pilot-scale roll press briquetting-CS-25 compactor/briquetter	Steam conditioning (25, 75, and 100^°^C), roll speed 1.5–2.3	Corn stover, 2.4 and 4 mm, 10 and 15% wb	139–160	422–481	NR	0.67–0.90	[3]
Pilot-scale roll press briquetting-CS-25 compactor/briquetter	Steam conditioning (25, 75, and 100^°^C), roll speed 1.3–2.5	Switchgrass, 2.4 and 4 mm, 10 and 15% wb,	184–220	351–527	NR	0.39–0.70	[3]
Pilot-scale conventional ring-die CPM Master model 818806 pelleting machine		Corn stover, 2.4 and 4 mm, 20% wb,	139–160	548–610	NR	0.94–0.95	[3]
Pilot-scale conventional ring-die CPM Master model 818806 pelleting machine		Switchgrass, 4 mm, 20% wb,	184–220	528–570	NR	0.75–0.86	[3]
Laboratory-scale CPM CL-5 pellet mill		Barely straw, 0.8–6.4 mm, 10% wb	96–180	NR	726–1033	0.49–0.98	[31]
Laboratory-scale CPM CL-5 pellet mill		Canola straw, 0.8–6.4 mm, 10% wb	144–247	NR	742–1015	0.22–0.98	[31]
Laboratory-scale CPM CL-5 pellet mill		Oat straw, 0.8–6.4 mm, 10% wb	111–196	NR	771–1002	0.44–0.99	[31]
Laboratory-scale CPM CL-5 pellet mill		Wheat straw, 0.8–6.4 mm, 10% wb	107–203	NR	760–1047	0.52–0.98	[31]
Ring-die pellet mill CPM Master model series 2000		Corn stover, 3.2–6.5 mm, 10% wb	50.9	469–625	529–843	0.96–0.98	[13, 23]
Ring-die pellet mill CPM Master model series 2000		Wheat straw, 3.2–6.5 mm, 10% wb	47.7	496–649	613–852	0.96–0.98	[13, 23]
Ring-die pellet mill CPM Master model series 2000		Big bluestem, 3.2–6.5 mm, 10% wb	46.6	467–648	517–778	0.96–0.98	[13, 23]
Ring-die pellet mill CPM Master model series 2000		Sorghum stalk, 3.2–6.5 mm, 10% wb	59.3	365–479	435–560	0.86–0.94	[13, 23]
Piston cylindercompression/densification apparatus, pressure applied through an Instron universal testing machine	100 and 150 MPa, preheating temperature 25–150^°^C	Corn stover, 2.4–4.6 mm, 7.3–15% wb	103–160	NR	825–1162	0.50–0.97	[21]
Piston cylindercompression/densification apparatus, pressure applied through an Instron universal testing machine	100 and 150 MPa, preheating temperature 25–150^°^C	Switchgrass, 2.4–4.6 mm, 9.2–15.1% wb	181–220	NR	417–1065	0–0.68	[21]
Pellet press (SPC 300)	Steam injection 2–6 kg/h	Fresh and 140 days stored sawdust from Scot Pine and Norway Spruce, 7.8–12.5% wb	NR	501–706	NR	0.79–0.99	[48]
Ultrasonic-vibration-assisted pelleting		Switchgrass, 1 mm, 15% wb	NR	415–560	NR	0.39–0.63	[49]

NR: not reported.

**Table 3:** Correlation coefficients.

Property	Fines	GMD	Lignin	Extractives	Moisture content
Bulk density	−0.03	−0.02	0.88	−0.49	0.57
Durability	−0.40	0.44	0.33	−0.15	0.03
